# The Mechanisms of Sevoflurane-Induced Neuroinflammation

**DOI:** 10.3389/fnagi.2021.717745

**Published:** 2021-08-05

**Authors:** Xiangfei Huang, Jun Ying, Danying Yang, Pu Fang, Xifeng Wang, Bin Zhou, Lieliang Zhang, Yang Fang, Wen Yu, Xing Liu, Qingcui Zhen, Fuzhou Hua

**Affiliations:** ^1^Department of Anesthesiology, The Second Affiliated Hospital of Nanchang University, Nanchang, China; ^2^Key Laboratory of Anesthesiology of Jiangxi Province, Nanchang, China; ^3^Department of Neurology, The First Affiliated Hospital of Nanchang University, Nanchang, China; ^4^Department of Anesthesiology, The First Affiliated Hospital of Nanchang University, Nanchang, China

**Keywords:** sevoflurane, neuroinflammation, microglia, blood–brain barrier, gut microbiota, cholinergic neurotransmission

## Abstract

Sevoflurane is one of the most commonly used inhaled anesthetics due to its low blood gas coefficient, fast onset, low airway irritation, and aromatic smell. However, recent studies have reported that sevoflurane exposure may have deleterious effects on cognitive function. Although neuroinflammation was most widely mentioned among the established mechanisms of sevoflurane-induced cognitive dysfunction, its upstream mechanisms have yet to be illustrated. Thus, we reviewed the relevant literature and discussed the most mentioned mechanisms, including the modulation of the microglial function, blood–brain barrier (BBB) breakdown, changes in gut microbiota, and ease of cholinergic neurotransmission to help us understand the properties of sevoflurane, providing us new perspectives for the prevention of sevoflurane-induced cognitive impairment.

## Introduction

Postoperative cognitive dysfunction, which has been included in the new conception of perioperative neurocognitive disorders, is a complication that occurs following surgery, especially in elderly people (>60 years old). It is worth noting that the trend for using anesthesia with surgery in elderly people is increasing (Miller et al., 2018). Among the anesthetics, sevoflurane has been widely used as an inhaled general anesthetic for anesthesia induction and maintenance in a variety of surgical treatments due to its low blood gas coefficient, fast onset, low airway irritation, and aromatic smell (Al Tmimi et al., 2015; Liu et al., 2015; Ye et al., 2016). In recent years, although sevoflurane was considered safe with a rapid clearance upon maintenance cessation, many studies have reported its capability to cause cognitive dysfunction (Fang et al., 2012; Lv et al., 2017; Zhu G. et al., 2017; Guo et al., 2018; Zhang et al., 2019).

To date, despite having various mechanisms of sevoflurane-induced cognitive dysfunction being mentioned by researchers, the exact pathophysiological changes remain unclear. Neuroinflammation, which was found to be associated with neurodegenerative diseases (Herranz et al., 2016; Melah et al., 2016), was found to be the most frequently mentioned mechanism. Several studies have shown that dysregulated neuroinflammation plays a key role in cognitive dysfunction (Cibelli et al., 2010; Terrando et al., 2011; Vacas et al., 2013; Riedel et al., 2014) through a variety of downstream mechanisms, including detrimental connectivity of brain network secondary to the dysfunction of synaptic transmission (Xiong et al., 2003), Tau phosphorylation, plaque formation, and dystrophic neurite growth (Streit et al., 2004). Due to this, sevoflurane might be one of the upstream causes of neuroinflammation (Zhang et al., 2013; Zheng et al., 2017; Cui et al., 2018), which can be exacerbated by other interferences such as sleep deprivation (Hou et al., 2019) and early-life adversity (Zhu Y. et al., 2017). However, the exact mechanisms underlying sevoflurane-induced neuroinflammation have yet to be elucidated.

Although the microglia were first recognized as immune surveillance cells of the central nervous system (CNS), recent studies have found them as the multifunctional cells that might interact with other CNS cells, which are associated with neurodegenerative diseases, such as Alzheimer’s disease (AD), Parkinson’s disease (PD), schizophrenia, autism, and multiple sclerosis (MS) (Prinz et al., 2019). Notably, one of the key features shared by these neurodegenerative diseases was microglia-mediated neuroinflammation. Concerning this, sevoflurane might induce the classical activation of microglia (M1), which is responsible for the production of proinflammatory cytokines, thereby reducing the alternative activation and acquired deactivation (M2), which are associated with anti-inflammation, reconstruction of extracellular matrix, and tissue repair (Tang and Le, 2016).

**FIGURE 1:**
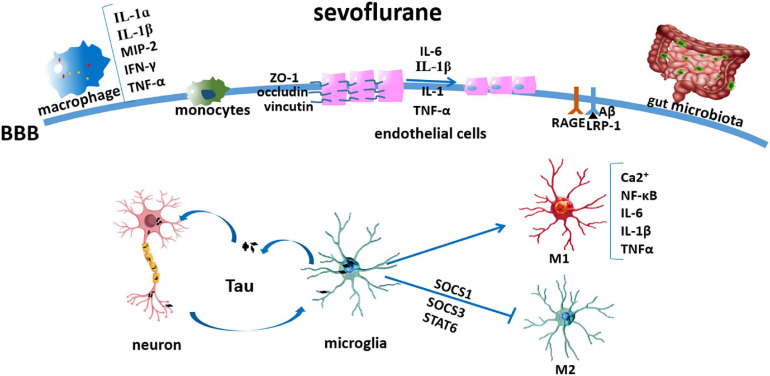
Sevoflurane modulating microglia and peripheral factors involved in neuroinflammation. Sevoflurane induced Tau transfer from the neurons to the microglia, thereby activating it to produce neurotoxic inflammatory molecules and cytokines, such as IL-1β and TNF-α; later, Tau can be phagocytized and secreted extracellularly and transmitted into the neuron again; sevoflurane induced microglia polarized into M1 activation *via* the NF-κB signaling pathway and inhibited its M2 activation by mediating SOCS1, SOCS3, and STAT6; sevoflurane induced macrophage inflammation, as shown by IL-1α, IL-1β, MIP-2, IFN-γ, and TNF-α increases; sevoflurane flattened the endothelial cell luminal surface and enlarged its perivascular spaces; and cytokines (IL-1β, IL-1, TNF-α, and IL-6) induced depression and redistribution of BBB adherens junction proteins (ZO-1, occludin, and vinculin); sevoflurane increased RAGE and decreased IRP-1 expressions; sevoflurane decreased intestinal microbiome abundance and diversity.

**FIGURE 2:**
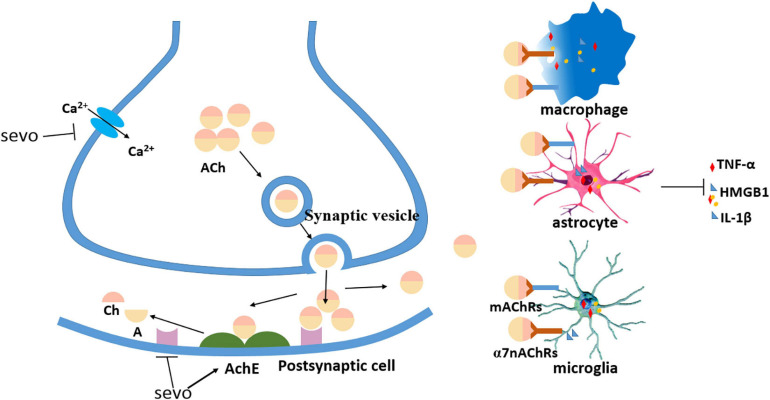
Sevoflurane decreased cholinergic neurotransmission. Sevoflurane partially decreased presynaptic acetylcholine (ACh) by inhibiting calcium currents, sevoflurane decreased postsynaptic ACh by increasing acetylcholinesterase (AChE), and sevoflurane reduced ACh binding with ACh receptors (AChRs) on the immune cells to inhibit the release of proinflammatory cytokines.

Aside from the inflammation initiated in the CNS, sevoflurane might also mediate the permeability of the blood–brain barrier (BBB) (Sun et al., 2019) and affect peripheral factors, such as peripheral immune cells and gut microbiota (Han et al., 2021), which have been widely explored in recent years (Figure 1). Another factor, the cholinergic synaptic transmission, has also been found to be involved in the inflammation modulation (Kalb et al., 2013), in which sevoflurane might play a proinflammatory role *via* the suppression of cholinergic neurotransmission (Yin et al., 2019; Figure 2).

In consideration of the clinical significance of sevoflurane, this study attempts to synthesize and discuss the mechanisms of sevoflurane-induced neuroinflammation, as all these mechanisms might be promising therapeutic targets that could be used to prevent, either partly or in total, neuroinflammation and consequent cognitive dysfunction.

## Mechanisms

### Activation and Regulation of Microglia and Peripheral Immune Cells

Prior to the use of the term “neuroinflammation,” immune responses have already been found to play a critical role in neurodegenerative diseases such as AD (McGeer et al., 1987; Griffin et al., 1989). Neurodegeneration is more aptly referred to the innate immune system in the brain rather than the adaptive immunity because leukocyte infiltration is limited. However, the intrinsic immune cells of the brain, especially microglia, are prominently activated in these situations (Streit et al., 2004). As early as 1932, the microglia were first mentioned by Pio del Río-Hortega to function as the macrophages of the CNS, but there have been few investigations followed up in the next 50 years using the limited methods that can distinguish microglia from other cells. Until 1995, microglia were known to be the vital components of the brain immune system (Streit and Kincaid-Colton, 1995). Specifically, they can be activated by adverse factors and are the first cells that appear to cause a neuroinflammatory response (Dheen et al., 2007). In fact, microglial activation is a hallmark of neuroinflammatory and neurodegenerative diseases, such as AD, PD, Huntington’s disease (HD), and amyotrophic lateral sclerosis (ALS) (Prinz et al., 2019).

Microglia can be polarized into two different activated states, namely, the classical activation (M1) that is characterized by proinflammation and production of reactive oxygen species, which is harmful to neurons, and the alternative activation (M2), which is typified by its opposite anti-inflammatory function and is helpful for tissue repair and wound healing (Boche et al., 2013). Sevoflurane can accelerate microglial migration, promote their activation, enhance their phagocytic efficiency, and promote their proliferation (Dang et al., 2018). In particular, (Zhang et al., 2013), reported that 4% sevoflurane for 6 h led to an increase of interleukin-6 (IL-6) *via* the nuclear factor-kappa B (NF-κB) pathway in isolated microglia (Zhang et al., 2013). The NF-κB signaling pathway has been well explored and is known to be associated with inflammation and cognitive impairment (Kaushal and Schlichter, 2008; Wang et al., 2014; Yang and Yuan, 2018), one of which is the microglial M1 activation (Kobayashi et al., 2013). Additionally, the activation of NF-κB signaling pathway can be induced by Ca^2+^ elevation, in which even the local Ca^2+^ transients may cause apparent NF-κB capacity (Meffert et al., 2003). Sevoflurane also increases the cytosolic calcium *via* the activation of inositol 1,4,5-trisphosphate receptors, leading to abnormal calcium release from the endoplasmic reticulum (Yang et al., 2008).

In contrast, sevoflurane not only enhances M1 polarization but also simultaneously suppresses M2 activation (Pei et al., 2017). Pei et al. (2017) found that in primary microglial cells isolated from the cerebral cortices of mice, the pretreatment of sevoflurane (2 or 4%) for 12 h before IL-4 (10 ng/ml) can suppress IL-4-induced increases of characteristic M2 marker genes Arg1, Ym1, and IL-10. The protein levels of the aforementioned genes were also inhibited by sevoflurane. Furthermore, other factors involved in M2 polarization, such as suppressor of cytokine signaling one upregulation, SOCS3 suppression, and signal transducer and activator of transcription 6 (STAT6) phosphorylation were found to be regulated by sevoflurane in a dose-dependent manner. In the same experiment, a similar conclusion was drawn in another model of human umbilical cord mesenchymal stromal cells–induced M2 polarization, in which sevoflurane attenuated Arg1, Ym1, and IL-10 protein and mRNA levels.

Moreover, both Tau and amyloid-beta (Aβ) peptides, as mentioned below, are canonical features of AD. Tau peptides can transfer between cells in a prion-like fashion, in which this seeding activity has been associated with AD progression (Laurent et al., 2018). Due to this, the relationship between microglia and the speed of Tau activity might be involved in a vicious cycle described as the following: first, Tau is transferred from the neurons to the microglia, thereby activating it to produce neurotoxic inflammatory molecules and cytokines, such as IL-1β and tumor necrosis factor-α (TNF-α); afterward, Tau can be phagocytized and secreted extracellularly, consequently increasing the progressive spread of tauopathy. Microglial phagocytosis further aggregates the spreading of Tau, given that the secreted Tau was easily transmissible to the neurons (Furman et al., 2017). Most recently, Dong et al. (2021) demonstrated that sevoflurane also induced Tau migration from the neurons to the microglia, leading to IL-6 generation and cognitive impairment. Therefore, the influence of sevoflurane on Tau migration might further worsen the neuroinflammation.

Previously, peripheral immune cells were thought to have less influence on the CNS due to the presence of the BBB; however, contradictory results have been found in models of patients with systemic inflammation and PD, in which monocytes are trafficked into the brain, influencing inflammatory CNS signaling (Zhou et al., 2009; Grozdanov et al., 2014). One of the specific mechanisms may be that proinflammatory factors break the BBB (reviewed in the section “Permeability Changes of Blood–Brain Barrier”).

### Permeability Changes of Blood–Brain Barrier

The BBB, formed with a monolayer of brain vascular endothelial cells (BVECs) and supported by astrocytes, pericytes, and a basement membrane, is essential for the regulation of the neural environment and brain homeostasis (Kniesel and Wolburg, 2000; Begley and Brightman, 2003; Abbott et al., 2010). The core element of the BBB is the formation of the blood vessel by the BVECs, acting as a physical barrier to control the microenvironment of cerebral cells. Unlike the peripheral tissue endothelium, BVECs in the brain are unique, as they are connected by numerous tight junctions that severely restrict ionic penetration and fluid movements (Abbott et al., 2006). The dysfunction of BBB is associated with a wide range of neurodegenerative diseases like AD, PD (Desai et al., 2007), and multiple system atrophy (Song et al., 2011).

Previous studies have focused on the protective role of sevoflurane in the BBB, but these articles were often in the precondition of adverse factors, such as surgery (Gao et al., 2019) or cerebral ischemia and reperfusion (Yu Q. et al., 2019). For example, Gao et al. (2019) reported that a short-time (i.e., 2 h) exposure to sevoflurane did not change cognitive impairment in rats; on the contrary, the Aβ, one of the main factors of postoperative cognitive dysfunction (Xu et al., 2014), was significantly eliminated by sevoflurane treatment *via* upregulating the expression of Aquaporin-4, a water channel expressed in astrocytic end feet. Additionally, Yu Q. et al. (2019) reported that sevoflurane improved the integrity of BBB after ischemia and reperfusion in rats, and they found that 1.5% of sevoflurane preconditioning induced migration of astrocyte toward infarct areas to form scaffolds facilitating neuroblast migration, and then subsequently promoted the formation of neural networks, which closely resemble those of the normal brain.

However, with regards to the aim of this article, there are also studies on sevoflurane-induced BBB disruption, which have mainly focused on BVECs. In particular, Acharya et al. (2015) found in an *in vivo* study of rats that exposure to 1–3% sevoflurane for 3 h resulted in the compromised BBB integrity and increased permeability, as shown by immunoglobulin G augmentation that leaked into the brain. Additionally, the structural changes in BBB-associated BVECs were observed in this study, resulting in a general flattening of the BVEC luminal surface and subsequent death. Similarly, Sun et al. (2019) observed enlarged perivascular spaces after the sevoflurane exposure in a duration-dependent manner, in which 78% of the capillaries were destroyed after 6 h of 2% sevoflurane treatment.

Although the astrocytes play a vital role in the maintenance of BBB integrity, there have been few studies attributing the disruption of sevoflurane-induced BBB to these astrocytes. It has been reported that the lengthy sevoflurane exposure disrupted the maturation of “tripartite synapse” by altering astrocyte morphogenesis and causing consequential behavioral deficits (Zhou et al., 2019). Despite the apparent adverse impact of sevoflurane on astrocytes, no researchers have linked this impact to direct BBB breakdown. More research is needed to clarify whether sevoflurane can damage BBB by regulating the function of astrocytes.

Interestingly, proinflammatory cytokines, such as IL-1 and TNF-α, can also induce BBB disruption, and their synergistic effects have also been reported (Quagliarello et al., 1991). As those tight junction proteins such as occludin play pivotal roles in maintaining the BBB integrity (Zehendner et al., 2011; Lv et al., 2018), TNF-α decreases the occludin in astrocytes through its specific effect on the TNF type-1 receptor *via* NF-κB activation (Wachtel et al., 2001). All these effects of TNF-α, such as the decreased levels of tight junction protein and the elevation of BBB permeability, have been attributed to the production of microvascular endothelial IL-6 (Rochfort et al., 2016). Similarly, IL-1β was found to induce neutrophil adhesion to the endothelial cells, decreasing the levels of occludin and zonula occludens protein 1 (ZO-1) and redistributing vinculin, another adherens junction protein, to enhance BBB permeability (Bolton et al., 1998). Thus, the breakdown of BBB may also still be the downstream mechanism of neuroinflammation, in turn, to further aggravate neuroinflammation.

Except for the structural breakdown of BBB, the receptors of the barrier, including receptors for advanced glycation end products (RAGE) and receptors for low-density lipoprotein receptor–related protein 1, play a vital role in the brain Aβ exchanges (Shibata et al., 2000; Deane et al., 2003; Herz and Marschang, 2003). More specifically, the expression of microglial RAGE enhanced the infiltration of astrocyte and microglial and increased the production of IL-1 and TNF-α (Fang et al., 2010). In particular, the study by Liu et al. (2013) proved that exposure to 2% sevoflurane for 2 h will lead to the increased RAGE and decreased IRP-1 in rats, possibly attributing that sevoflurane might induce neuroinflammation by regulating the expressions of RAGE and IRP-1, ultimately leading to Aβ and cytokine accumulation in the brain.

### Decreased Gut Microbiota

The dysregulation of gut microbe might also influence the brain through immune molecules, hormones, and relevant metabolites (Mayer et al., 2015; Smith, 2015), such as impairing BBB function by regulating tight junction proteins (Braniste et al., 2014), modulating brain development and behavior by altering canonical signaling pathways, expressing synaptic plasticity–related proteins and allowing neurotransmitter turnover (Diaz Heijtz et al., 2011). The correlations between gut microbes and human neurological diseases, such as MS, PD, AD, HD, ALS, and neuromyelitis optica spectrum disorders, have been well illustrated in previous studies (Tremlett et al., 2017). The gastrointestinal microbiota were further proven to be a key neuroinflammation modulator, possibly by recruiting monocytes into the brain and/or modulating the stress response *via* the regulation of the activity of the hypothalamic–pituitary–adrenal axis (Rea et al., 2016). Given that gut microbes have been well explored in human neurological diseases and Rea et al. (2016) have reviewed the relationship between gut microbiome and neuroinflammation, the role of gut microbes in regulating neuroinflammation may be compelling.

Frohlich et al. (2016) performed an *in vivo* study in rats that the short-term antibiotic use (mixed antibiotics of ampicillin, bacitracin, meropenem, neomycin, and vancomycin at 8–11 weeks of age for 11 days) significantly disrupted the gut bacterial community and impaired the cognitive performance. Although researchers have discussed the antibacterial activity of the halogenated anesthetic decades ago, these were always studied at very high concentrations (Wardley-Smith and Nunn, 1971; Molliex et al., 1998), which carries no actual clinical significance. Until recently, a new study performed by Han et al. (2021) finally gave us a certain answer, showing that a clinical concentration of sevoflurane did change the gut microbiota. More specifically, 1.3 minimum alveolar concentration (MAC) of sevoflurane gas (i.e., 2.21%) for 4 h was found to have decreased the diversity and abundance of the intestinal microbiome. Similarly, a previous study conducted on isoflurane proved that one MAC isoflurane for 4 h in juvenile rats (postnatal Day 7) significantly changed their gut microbiota in the long term (i.e., 42 days), in which such changes may be the potential mechanism of isoflurane-induced cognitive dysfunction (Wang et al., 2019).

It is worth noting that Chamberlain et al. (2017) provided a new perspective on the influence of sevoflurane on bacterial behavior, revealing that sevoflurane at clinically relevant concentrations (i.e., 1.5% and 3%) reduced *Escherichia coli* and *Pseudomonas aeruginosa* swimming, and conversely enhanced the biofilm formation of *Staphylococcus aureus* and *Enterococcus faecalis*, which are the important behaviors in bacteria for liquid mobility and consequent antibiotic resistance. This study also provides a new orientation to evaluate the potential impact of sevoflurane on gut bacteria, rather than focusing on the abundance and diversity of the intestinal microbiome. Unfortunately, neither sevoflurane nor isoflurane experiments have been further extended to the regulation of neuroinflammation after the disturbance of the intestinal microbiome. This should be one of the key directions for future research on neuroinflammation caused by the inhaled anesthetic.

Although there are few direct studies on the regulation of the intestinal microbiome by sevoflurane, in line with the results of the isoflurane study, the effect of sevoflurane on reducing the diversity and abundance of the intestinal microbiome may be convincing. According to the existing studies, when exploring the mechanism of sevoflurane-induced neuroinflammation, it may be safe to put forward the influence of the gut microbiota–brain axis, though more follow-up experiments are needed to prove these findings further.

### Modulation of Cholinergic Synaptic Transmission

The selected cholinergic neuron loss in the canonical neuroinflammation-related disease, AD, has been observed decades ago (Davies and Maloney, 1976). The principal neurotransmitter of the vagus nerve is acetylcholine (ACh), which is associated with cognition and learning ability (Conner et al., 2003; Gotti et al., 2006; Lima et al., 2014). There are two types of ACh receptors (AChRs), namely, the muscarinic receptor (mAChR) and nicotinic receptor (nAChR), which are distributed in both the CNS and periphery. Although the ACh anti-inflammatory function was mostly found with the nAChRs, or more specifically, the subdivided α7 nicotinic acetylcholine receptors (α7nAChRs) (Pavlov and Tracey, 2005), the contribution of mAChR has also been reported (Fujii et al., 2017). The core of the cholinergic anti-inflammatory pathway is the α7nAChRs, which are expressed on the surfaces of the immune cell and are known to be required for the cholinergic inhibition of TNF and other proinflammatory cytokines, such as IL-1β and high-mobility group protein B1 (Wang et al., 2003, 2004). Peripheral immune cells such as macrocytes and cerebral immune cells, such as microglia and astrocytes, all have α7nAChRs, in which ACh binding can reduce the production of proinflammatory cytokine.

In addition, it is clear that anti-inflammation and pro-resolution are not equivalent processes. Inflammation resolution, involving pro-resolving mediators, is also vital for the tissues affected by inflammation to regain function (Serhan and Savill, 2005). Meanwhile, Mirakaj et al. (2014) reported that the resolution and pro-resolving mediators of inflammation were controlled by the vagus nerve. Although the vagus nerve induced the anti-inflammatory pathway and pro-resolution function might be vital for neuroinflammation prevention, ACh transmission might also be inhibited by sevoflurane, and the potential mechanisms are reviewed as follows.

It has been reported that the clinical concentration of sevoflurane can inhibit ACh release in a dose-dependent manner (Shichino et al., 1998). Furthermore, an experiment by Chen et al. (2015) in *Drosophila melanogaster* showed that 1, 2, and 3% sevoflurane for 24 h reduced presynaptic cholinergic neurotransmission, partially by inhibiting calcium current. Interestingly, the *in vitro* study by Naruo et al. (2005) demonstrated that a clinically relevant sevoflurane concentration (i.e., 0.5–3%) inhibited the postsynaptic cholinergic neurotransmitters without the influence of presynaptic exocytosis or endocytosis, which instead functioned by nAChR blocking on the postsynaptic neuron. Despite these findings, the exact mechanism by which ACh moves to the α7nAChRs on immune cells remains to be elucidated, and it has been postulated that the ACh diffuses to α7nAChRs following its release from the vagus nerve axon terminals. Thus, supposing that this postulate is true, nAChR blockage on the postsynaptic neuron *via* sevoflurane induction increases ACh diffusion to the immune cells. However, this effect might be insignificant because aside from decreasing presynaptic and postsynaptic cholinergic neurotransmitters, sevoflurane might also increase acetylcholinesterase (AChE) activity and expression (Yin et al., 2019). In consideration of the rapid AChE catabolism, we assumed that ACh redundancy caused by the nAChR blockage on the postsynaptic neuron might not have influenced the diffused quantity, although more studies are needed to verify this hypothesis.

## Discussion

In consideration of the clinical importance of sevoflurane, understanding its properties and figuring out its potential noxious effects are significant. Although numerous studies have explored the mechanism of sevoflurane-induced cognitive dysfunction and have brought up multiple mechanisms to support these hypotheses, neuroinflammation was the most mentioned in recent years. Meanwhile, our understanding of the upstream mechanisms of how sevoflurane induces neuroinflammation was enriched with time. Aside from cerebral tissue–initiated inflammation, BBB breakdown, modulation of cholinergic anti-inflammatory pathway, the influence of sevoflurane on peripheral factors and gut microbiota was also investigated. Although the mechanisms of sevoflurane induced-neuroinflammation might not limit to the aforementioned mechanisms, they should still be promising orientations for researchers to understand the impact of sevoflurane on cognitive function well.

In fact, the effects of sevoflurane on neuroinflammation remain controversial. In addition to its proinflammatory function, this anesthetic was also shown to reduce neuroinflammation caused by adverse effects, such as brain ischemic diseases, which was its most evidenced benefit. For instance, the study by Hwang et al. (2017) showed that sevoflurane postconditioning decreased the production of multiple proinflammatory cytokines such as TNF-α, IL-6, and IL-1β, *via* the Toll-like receptor-4/NF-κB pathway, which was activated by cerebral ischemia/reperfusion injury. Similarly, Xue et al. (2020) reported that sevoflurane postconditioning significantly attenuated the activation of hypoxic–ischemic brain injury–induced microglia/macrophage and impairment of the cognitive function.

In line with this, sevoflurane might also play a dual role in microglial activation, in which Xie et al., found that sevoflurane treatment increased expressions of NF-κB and cytokine IL-6 in isolated neuroglioma cells (Zhang et al., 2013); however, Yu X. et al. (2019) observed different results, in which sevoflurane reduced NF-κB expression and production of proinflammatory cytokines following the lipopolysaccharide (LPS) treatment. These contradictory results might be caused by the concentration and/or duration differences between the studies. In the study by Xie et al., 4.1% sevoflurane for 6 h was used, whereas 3.3% sevoflurane for l h in the study by Yu et al., was only used. Similarly, TNF-α decrease was found in resting isolated microglia after 2% sevoflurane treatment, but a significant increase was observed after 4% sevoflurane. Another similar result was also found in LPS-activated microglia, in which 4% sevoflurane increased IL-1β and IL-6, but this was not observed on using 2% sevoflurane (Ye et al., 2013). This concentration-dependent and/or duration-dependent phenomenon was also observed in the effect of sevoflurane on peripheral immune cells. It is apparent to see that the suppressive effect of sevoflurane on peripheral immune cells was found at relatively low concentrations, which, in most cases, was less than 3% (Mitsuhata et al., 1995; Wada et al., 2007; Kong et al., 2010). In contrast, its stimulative function is always observed in relatively high concentrations, such as two MAC in rats (Morisaki et al., 1997; Kotani et al., 1999) and 5% in humans (Morisaki et al., 1998).

The gut microbes were well explored in human neurological diseases, such as MS, PD, AD, HD, and ALS (Tremlett et al., 2017). Furthermore, the Rea et al. (2016) reviewed the relationship between gut microbiome and neuroinflammation. The gut microbiome–neuroinflammation process might partly be bridged by the breakdown of BBB. For example, the study by Braniste et al. (2014) showed that the lack of gut microbiota was associated with lower expression of the tight junction proteins (e.g., occludin, ZO-1, and claudin-5) and increased the permeability of BBB subsequently. As we reviewed in the section “Permeability Changes of Blood–Brain Barrier,” abundant studies confirmed that sevoflurane induced the breakdown of BBB to cause neuroinflammation. Unfortunately, to date, there is only one study published this year (Han et al., 2021) which reported that sevoflurane might decrease the diversity and abundance of the intestinal microbiome, and this study only focused on the effect of sevoflurane on intestinal bacteria yet did not extend to downstream neuroinflammation. Even so, in line with the similar conclusion drawn by a previous isoflurane study (Wang et al., 2019), the regulative function of sevoflurane on gut bacteria seems convincing. Although the evidence is not particularly strong at present, sevoflurane may be a promising area of research considering the regulation of neuroinflammation by intestinal flora, and further studies are needed to fill in this gap.

It is worth noting that the resulting increase in inflammatory cytokines can increase the permeability of BBB and in turn aggravate neuroinflammation (Quagliarello et al., 1991). Similarly, more research is needed to confirm whether the decrease in the intestinal flora caused by sevoflurane was mediated by neuroinflammation. After all, the brain–gut axis was known as a bidirectional axis (Dinan and Cryan, 2017). This means that the breakdown of BBB and intestinal flora disorder may also be the result of the occurrence of neuroinflammation, which in turn further aggravates neuroinflammation.

Although the inhibitory effect of sevoflurane on the cholinergic nerve is well described in multiple studies, different findings can still be seen. Silva et al. (2005) conversely reported that sevoflurane increased the ACh release, and his opinion was enforced by similar findings in other volatile anesthetics, such as halothane (Gomez et al., 1999; Adachi et al., 2002) and isoflurane (Gomez et al., 2000). This discrepancy might be explained by the differences in the models and drug administration, which was observed in an *in vivo* study with 0.5 MAC (i.e., 1.1%) to 1.5 MAC (i.e., 3.3%) of sevoflurane gas (Shichino et al., 1998); however, the study by Silva et al. (2005) and relevant isoflurane studies were performed *in vitro*. As previously mentioned, sevoflurane partially reduced cholinergic neurotransmissions by blockage of the postsynaptic nAChRs, but the question remains on whether the anesthetic also directly blocks the α7nAChRs on the immune cells. This is an interesting question to explore in future studies.

Sevoflurane might induce neuroinflammation through complicated and interacting mechanisms, in which its proinflammatory function is dependent on concentration and duration under certain pathological models. However, the neuroprotective function of sevoflurane should also not be ignored. Therefore, future studies with uniform models should find the thresholds of both the proinflammatory and anti-inflammatory functions of sevoflurane, which will be more instructive for its clinical use.

## Author Contributions

XH and DY reviewed the literature and drafted the manuscript. FH, JY, and PF participated in the design and coordination of the review. XW, BZ, and LZ participated in the preparation of the manuscript. YF, WY, XL, and QZ participated in the review of the literature. All authors read and approved the final manuscript.

## Conflict of Interest

The authors declare that the research was conducted in the absence of any commercial or financial relationships that could be construed as a potential conflict of interest.

## Publisher’s Note

All claims expressed in this article are solely those of the authors and do not necessarily represent those of their affiliated organizations, or those of the publisher, the editors and the reviewers. Any product that may be evaluated in this article, or claim that may be made by its manufacturer, is not guaranteed or endorsed by the publisher.
